# Use of CMR imaging to assess the effect of lipoprotein apheresis in patients with refractory angina and raised lipoprotein(a)

**DOI:** 10.1186/1532-429X-17-S1-P137

**Published:** 2015-02-03

**Authors:** Tina Khan, Ricardo Wage, Mahmoud Barbir, Dudley J Pennell

**Affiliations:** Royal Brompton and Harefield NHS Foundation Trust, London, UK

## Background

Angina which is refractory to conventional medical therapy and revascularisation with bypass surgery and/or percutaneous coronary intervention is extremely challenging to manage with limited treatment options and a pressing need to establish effective therapy.

Lipoprotein(a) [Lp(a)], a genetically determined form of LDL-cholesterol, is an independent cardiovascular risk factor which is felt to increase risk by promoting thrombosis; enhancing intimal lipoprotein deposition, and by potentially affecting myocardial perfusion, micro-vascular function, plasma viscosity and endothelial function. Lipoprotein apheresis is currently the most effective treatment available for raised Lp(a). Increasing evidence suggests that aggressive Lp(a) reduction may improve cardiovascular outcomes, although much more prospective research is required to confirm this.

## Methods

We are conducting a prospective, blinded randomised controlled cross-over study of patients with refractory angina and raised Lp(a), randomised to undergoing lipoprotein apheresis or ‘sham' apheresis with assessment of the impact of treatment on myocardial perfusion, carotid atherosclerosis, endothelial function, thrombogenesis, oxidised phospholipids, exercise capacity, angina and quality of life.

Our primary hypothesis is that lipoprotein apheresis improves quantitative myocardial perfusion assessed by Myocardial Perfusion Reserve (MPR) detected by stress/rest Cardiovascular Magnetic Resonance (CMR), in patients with refractory angina and raised Lp(a).

At least 20 patients with refractory angina and elevated Lp(a) will be recruited and randomly assigned to either a treatment arm who will undergo lipoprotein apheresis weekly for three months, or to a control group who will receive placebo ‘sham' sessions weekly for three months. All patients will have CMR at baseline assessing functional parameters, first-pass quantitative perfusion at stress and rest and late gadolinium enhancement (LGE), and quantitative assessment of carotid atherosclerosis. Following the three month period of either active or sham therapy all baseline tests will be repeated to determine the net treatment effect. After a wash-out period of at least 1 month, patients will cross-over to the opposite treatment arm with the process repeated.

## Results

The trial is currently in progress. Analysis of quantitative myocardial perfusion and carotid indices will be performed fully blinded with results expected by the end of 2015.

## Conclusions

We are conducting a randomised controlled cross-over trial using CMR to assess whether lipoprotein apheresis is beneficial in patients with refractory angina and raised Lp(a). Our research will confirm whether raised Lp(a) should be a therapeutic target in patients with refractory angina and will provide valuable insights into the mechanistic role of Lp(a) in cardiovascular disease.

## Funding

This project is supported by the NIHR Cardiovascular Biomedical Research Unit of Royal Brompton and Harefield NHS Foundation Trust and Imperial College London. Apheresis equipment being used has been provided by Kaneka Pharma.

